# Empty pelvis syndrome: PelvEx Collaborative guideline proposal

**DOI:** 10.1093/bjs/znad301

**Published:** 2023-09-26

**Authors:** PelvEx Collaborative, C T West, M A West, A H Mirnezami, I Drami, A Denys, T Glyn, P A Sutton, J Tiernan, C Behrenbruch, G Guerra, P S Waters, N Woodward, S Applin, S J Charles, S A Rose, E Pape, G H van Ramshorst, A G J Aalbers, Aziz N Abdul, N Abecasis, M Abraham-Nordling, T Akiyoshi, R Alahmadi, W Alberda, M Albert, M Andric, M Angeles, E Angenete, A Antoniou, J Armitage, R Auer, K K Austin, E Aytac, O Aziz, N Bacalbasa, R P Baker, M Bali, S Baransi, G Baseckas, B Bebington, M Bedford, B K Bednarski, G L Beets, P L Berg, C Bergzoll, S Biondo, K Boyle, L Bordeianou, E Brecelj, A B Bremers, K Brown, M Brunner, P Buchwald, A Bui, A Burgess, J W A Burger, D Burling, E Burns, N Campain, S Carvalhal, L Castro, A Caycedo-Marulanda, W Ceelen, K K L Chan, G J Chang, M H Chew, A K Chok, P Chong, H K Christensen, H Clouston, D Collins, A J Colquhoun, J Constantinides, A Corr, M Coscia, M Cosimelli, C Cotsoglou, P E Coyne, R S Croner, L Damjanovic, I R Daniels, M Davies, R J Davies, C P Delaney, J H W de Wilt, Q D Denost, C Deutsch, D Dietz, S Domingo, E J Dozois, E Drozdov, M Duff, E Egger, T Eglinton, J M Enrique-Navascues, E Espín-Basany, M D Evans, B Eyjólfsdóttir, M Fahy, N S Fearnhead, S Fichtner-Feigl, K Flatmark, F Fleming, B Flor, J Folkesson, K Foskett, F A Frizelle, J Funder, M A Gallego, E García-Granero, J L García-Sabrido, M Gargiulo, V G Gava, L Gentilini, M L George, V George, P Georgiou, A Ghosh, L Ghouti, A Gil-Moreno, F Giner, N Ginther, T Glover, P Goffredo, T Golda, C M Gomez, B Griffiths, F Gwenaël, C Harris, D A Harris, J A W Hagemans, V Hanchanale, D P Harji, C Helbren, R M Helewa, G Hellawell, A G Heriot, D Hochman, W Hohenberger, T Holm, A Holmström, R Hompes, B Hornung, S Hurton, E Hyun, M Ito, L H Iversen, J T Jenkins, K Jourand, S Kaffenberger, G V Kandaswamy, S Kapur, Y Kanemitsu, M Kaufman, M Kazi, S R Kelley, D S Keller, M E Kelly, S Kersting, S H J Ketelaers, M S Khan, J Khaw, H Kim, H J Kim, R Kiran, C E Koh, N F M Kok, R Kokelaar, C Kontovounisios, F Kose, M Koutra, M Kraft, H Ø Kristensen, S Kumar, M Kusters, V Lago, Z Lakkis, B Lampe, M C Langheinrich, T Larach, S G Larsen, D W Larson, W L Law, S Laurberg, P J Lee, M Limbert, A Loria, M L Lydrup, A Lyons, A C Lynch, M Mackintosh, C Mann, C Mantyh, K L Mathis, C F S Margues, A Martinez, A Martling, W J H J Meijerink, A Merchea, S Merkel, A M Mehta, D R McArthur, J J McCormick, F D McDermott, J S McGrath, A McPhee, J Maciel, S Malde, S Manfredelli, S Mikalauskas, D Modest, J R T Monson, J R Morton, T G Mullaney, A S Navarro, H Neeff, I Negoi, J W M Neto, B Nguyen, M B Nielsen, G A P Nieuwenhuijzen, P J Nilsson, S Nordkamp, S T O’Dwyer, K Paarnio, G Palmer, E Pappou, J Park, D Patsouras, A Peacock, G Pellino, A C Peterson, F Pfeffer, F Piqeur, J Pinson, G Poggioli, D Proud, M Quinn, A Oliver, A Quyn, R W Radwan, N Rajendran, C Rao, S Rasheed, P C Rasmussen, E Rausa, S E Regenbogen, H M Reims, A Renehan, J Rintala, R Rocha, M Rochester, J Rohila, J Rothbarth, M Rottoli, C Roxburgh, H J T Rutten, B Safar, P M Sagar, A Sahai, A Saklani, T Sammour, R Sayyed, A M P Schizas, E Schwarzkopf, D Scripcariu, V Scripcariu, G Seifert, C Selvasekar, M Shaban, I Shaikh, D Shida, A Simpson, T Skeie-Jensen, N J Smart, P Smart, J J Smith, T Smith, A M Solbakken, M J Solomon, M M Sørensen, M Spasojevic, S R Steele, D Steffens, K Stitzenberg, L Stocchi, N A Stylianides, T Swartling, H Sumrien, T Swartking, H Takala, E J Tan, C Taylor, D Taylor, P Tejedor, A Tekin, P P Tekkis, J Teras, M R Thanapal, H V Thaysen, E Thorgersen, R Thurairaja, E L Toh, P Tsarkov, J Tolenaar, Y Tsukada, S Tsukamoto, J J Tuech, G Turner, W H Turner, J B Tuynman, M Valente, J van Rees, D van Zoggel, W Vásquez-Jiménez, C Verhoef, M Vierimaa, G Vizzielli, E L K Voogt, K Uehara, C Wakeman, S Warrier, H H Wasmuth, K Weber, M R Weiser, O L Westney, J M D Wheeler, J Wild, M Wilson, A Wolthuis, H Yano, B Yip, J Yip, R N Yoo, M A Zappa, D C Winter

Empty pelvis syndrome is a complex challenge resulting from the dead space generated by exenteration. A systematic review of reconstructive techniques to prevent empty pelvis syndrome cited inconsistent definitions and heterogeneous outcome reporting, and was therefore unable to make strong conclusions in favour of a particular strategy^1^. The severity of complications escalates with more radical surgery as boundaries are pushed^2,3^. The guidelines proposed here will establish standardized definitions, outcomes, descriptors, patient perspectives, and unmet needs.

The study will be conducted on behalf of the PelvEx Collaborative, an international group of 140 units from around the world; see the Collaborators section. Diverse patient representation will be sought in liaison with Bowel Research UK, CommunitiesFirst, and the World Federation of Incontinence and Pelvic Problems. The design is a three-stage consensus study design using Core Outcome Measures in Effectiveness Trials (COMET) and COnsensus-based Standards for the selection of health Measurement INstruments (COSMIN) guidance.

## Collaborators

**PelvEx Collaborative**

West CT, West MA, Mirnezami AH, Drami I, Denys A, Glyn T, Sutton PA, Tiernan J, Behrenbruch C, Guerra G, Waters PS, Woodward N, Applin S, Charles SJ, Rose SA, Pape E, van Ramshorst GH, Aalbers AGJ, Abdul Aziz N, Abecasis N, Abraham-Nordling M, Akiyoshi T, Alahmadi R, Alberda W, Albert M, Andric M, Angeles M, Angenete E, Antoniou A, Armitage J, Auer R, Austin KK, Aytac E, Aziz O, Bacalbasa N, Baker RP, Bali M, Baransi S, Baseckas G, Bebington B, Bedford M, Bednarski BK, Beets GL, Berg PL, Bergzoll C, Biondo S, Boyle K, Bordeianou L, Brecelj E, Bremers AB, Brown K, Brunner M, Buchwald P, Bui A, Burgess A, Burger JWA, Burling D, Burns E, Campain N, Carvalhal S, Castro L, Caycedo-Marulanda A, Ceelen W, Chan KKL, Chang GJ, Chew MH, Chok AK, Chong P, Christensen HK, Clouston H, Collins D, Colquhoun AJ, Constantinides J, Corr A, Coscia M, Cosimelli M, Cotsoglou C, Coyne PE, Croner RS, Damjanovic L, Daniels IR, Davies M, Davies RJ, Delaney CP, de Wilt JHW, Denost QD, Deutsch C, Dietz D, Domingo S, Dozois EJ, Drozdov E, Duff M, Egger E, Eglinton T, Enrique-Navascues JM, Espín-Basany E, Evans MD, Eyjólfsdóttir B, Fahy M, Fearnhead NS, Fichtner-Feigl S, Flatmark K, Fleming F, Flor B, Folkesson J, Foskett K, Frizelle FA, Funder J, Gallego MA, García-Granero E, García-Sabrido JL, Gargiulo M, Gava VG, Gentilini L, George ML, George V, Georgiou P, Ghosh A, Ghouti L, Gil-Moreno A, Giner F, Ginther N, Glover T, Goffredo P, Golda T, Gomez CM, Griffiths B, Gwenaël F, Harris C, Harris DA, Hagemans JAW, Hanchanale V, Harji DP, Helbren C, Helewa RM, Hellawell G, Heriot AG, Hochman D, Hohenberger W, Holm T, Holmström A, Hompes R, Hornung B, Hurton S, Hyun E, Ito M, Iversen LH, Jenkins JT, Jourand K, Kaffenberger S, Kandaswamy GV, Kapur S, Kanemitsu Y, Kaufman M, Kazi M, Kelley SR, Keller DS, Kelly ME, Kersting S, Ketelaers SHJ, Khan MS, Khaw J, Kim H, Kim HJ, Kiran R, Koh CE, Kok NFM, Kokelaar R, Kontovounisios C, Kose F, Koutra M, Kraft M, Kristensen HØ, Kumar S, Kusters M, Lago V, Lakkis Z, Lampe B, Langheinrich MC, Larach T, Larsen SG, Larson DW, Law WL, Laurberg S, Lee PJ, Limbert M, Loria A, Lydrup ML, Lyons A, Lynch AC, Mackintosh M, Mann C, Mantyh C, Mathis KL, Margues CFS, Martinez A, Martling A, Meijerink WJHJ, Merchea A, Merkel S, Mehta AM, McArthur DR, McCormick JJ, McDermott FD, McGrath JS, McPhee A, Maciel J, Malde S, Manfredelli S, Mikalauskas S, Modest D, Monson JRT, Morton JR, Mullaney TG, Navarro AS, Neeff H, Negoi I, Neto JWM, Nguyen B, Nielsen MB, Nieuwenhuijzen GAP, Nilsson PJ, Nordkamp S, O’Dwyer ST, Paarnio K, Palmer G, Pappou E, Park J, Patsouras D, Peacock A, Pellino G, Peterson AC, Pfeffer F, Piqeur F, Pinson J, Poggioli G, Proud D, Quinn M, Oliver A, Quyn A, Radwan RW, Rajendran N, Rao C, Rasheed S, Rasmussen PC, Rausa E, Regenbogen SE, Reims HM, Renehan A, Rintala J, Rocha R, Rochester M, Rohila J, Rothbarth J, Rottoli M, Roxburgh C, Rutten HJT, Safar B, Sagar PM, Sahai A, Saklani A, Sammour T, Sayyed R, Schizas AMP, Schwarzkopf E, Scripcariu D, Scripcariu V, Seifert G, Selvasekar C, Shaban M, Shaikh I, Shida D, Simpson A, Skeie-Jensen T, Smart NJ, Smart P, Smith JJ, Smith T, Solbakken AM, Solomon MJ, Sørensen MM, Spasojevic M, Steele SR, Steffens D, Stitzenberg K, Stocchi L, Stylianides NA, Swartling T, Sumrien H, Swartking T, Takala H, Tan EJ, Taylor C, Taylor D, Tejedor P, Tekin A, Tekkis PP, Teras J, Thanapal MR, Thaysen HV, Thorgersen E, Thurairaja R, Toh EL, Tsarkov P, Tolenaar J, Tsukada Y, Tsukamoto S, Tuech JJ, Turner G, Turner WH, Tuynman JB, Valente M, van Rees J, van Zoggel D, Vásquez-Jiménez W, Verhoef C, Vierimaa M, Vizzielli G, Voogt ELK, Uehara K, Wakeman C, Warrier S, Wasmuth HH, Weber K, Weiser MR, Westney OL, Wheeler JMD, Wild J, Wilson M, Wolthuis A, Yano H, Yip B, Yip J, Yoo RN, Zappa MA, Winter DC.

## Data Availability

Not applicable.
